# Update on glaucoma

**Published:** 2022-01-31

**Authors:** Elmien Wolvaardt, Victor H Hu

**Affiliations:** 1 Editor: *Community Eye Health Journal*, International Centre for Eye Health, London School of Hygiene & Tropical Medicine, London, UK.; 2 Assistant Clinical Professor: International Centre for Eye Health, London School of Hygiene & Tropical Medicine and Consultant Ophthalmologist, Mid Cheshire NHS Hospitals, UK.

**Glaucoma affects millions of people worldwide, but it is difficult to diagnose and manage.**

**Figure:**
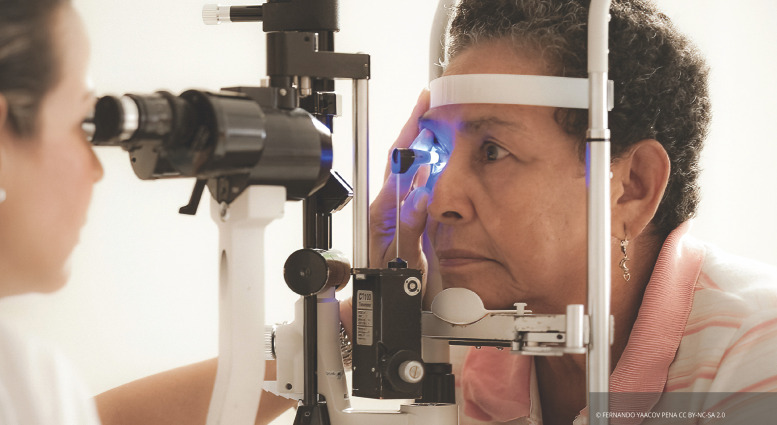
Screening for glaucoma. **COLOMBIA**

Glaucoma is a major cause of irreversible blindness worldwide, and it also causes substantial disability before patients become blind. Glaucoma is difficult to detect and diagnose, and it is highly undertreated globally. In most surveys carried out in high-income countries, over 50% of people found to have glaucoma had not been diagnosed and are therefore not receiving treatment, rising to over 90% in low- and/or middle-income countries. This is because glaucoma is mostly asymptomatic until relatively late in the disease, so patients do not notice that there is a problem. In many low- and/or middle-income settings, as many as 35% of people diagnosed with glaucoma already have severe sight loss: they presented too late to benefit from interventions that may have preserved their vision.

Our aim in this issue of the *Community Eye Health Journal* is to provide practical articles that will help clinicians facing the challenge of providing care for glaucoma patients. Topics include glaucoma detection and diagnosis, gonioscopy (a vital examination technique), the latest guidelines for open-angle glaucoma management, and tips for managing neovascular glaucoma and the painful, blind eye. We also look at the importance of counselling and how low vision support can benefit patients with vision loss due to glaucoma.

Glaucoma is a huge topic, and this issue covers care for adults only, concentrating on open-angle disease. Many useful resources are listed, including an additional 10 pages of new content only available on our website and smartphone app (see back page for details).

Our first *Community Eye Health Journal* webinar will take place on World Glaucoma Day, 22 March 2022, featuring a number of authors who have contributed to this issue. Subscribe to our mailing list (**www.cehjournal.org/subscribe**) to receive the link for the webinar and updates when new articles are published online.

